# Assessment of Interleukin-6 (IL-6) and Cotinine Levels in the Saliva of Patients with Precancerous Lesions and Oral Mucosa Cancer

**DOI:** 10.3390/jcm15072684

**Published:** 2026-04-02

**Authors:** Iwona Niedzielska, Jacek Kasperski, Karolina Pałkiewicz-Gierka, Barbara Trepka-Sirek, Zbigniew Puszczewicz

**Affiliations:** 1 Department of Cranio-Maxillo-Facial Surgery, Medical University of Silesia, 40-027 Katowice, Poland; 2 Department of Dental Prosthetics, Division of Medical Sciences in Zabrze, Medical University of Silesia in Katowice, 15 Poniatowskiego Street, 40-055 Katowice, Poland; protstom@sum.edu.pl

**Keywords:** salivary biomarkers, IL-6, cotinine, oral cancer, precancerous oral lesions, smoking, alcohol consumption, TNM classification, inflammation, early diagnosis

## Abstract

**Background/Objectives:** Oral squamous cell carcinoma (OSCC) and oral potentially malignant disorders (OPMDs) remain major clinical challenges due to late diagnosis and limited prognostic markers. This study evaluated salivary interleukin-6 (IL-6) and cotinine as potential biomarkers for oral epithelial transformation and exposure to risk factors. **Methods:** From 267 individuals with histopathologically confirmed OPMDs or OSCC, 47 patients met the inclusion criteria and were enrolled (18 OPMDs, 29 OSCC). A control group comprised 40 individuals without oral mucosal pathology. Unstimulated saliva samples were collected and analyzed for IL-6 and cotinine concentrations. Biomarker levels were compared among groups and evaluated in relation to smoking exposure, alcohol use, and clinicopathological parameters. **Results:** Salivary IL-6 and cotinine levels differed significantly among groups (*p* < 0.05), with the highest concentrations observed in OSCC patients, intermediate levels in OPMDs, and the lowest levels in controls. Cotinine levels were significantly higher in smokers and individuals with greater tobacco exposure in both study groups, whereas IL-6 concentrations were not significantly associated with smoking or alcohol consumption. No correlation between IL-6 and cotinine was found in OPMDs or OSCC; however, a moderate negative correlation was observed in controls. **Conclusions:** Salivary IL-6 and cotinine demonstrate potential as complementary, non-invasive biomarkers for assessing oral epithelial transformation and tobacco exposure. Their combined evaluation may support risk stratification and early detection in patients with OPMDs.

## 1. Introduction

Oral squamous cell carcinoma (OSCC) is a prevalent malignancy of the head and neck region, associated with high morbidity and persistently low five-year survival rates. Late diagnosis remains a major clinical challenge, often limiting treatment effectiveness and worsening patient prognosis [1,2]. Therefore, early detection of premalignant lesions and identification of reliable biomarkers for malignant transformation are critical.

Most OSCC cases develop from oral potentially malignant disorders (OPMDs), a group of lesions and conditions defined by the World Health Organization (WHO) as carrying an increased risk of malignant transformation. Common OPMDs include leukoplakia, erythroplakia, and mixed forms such as erythroleukoplakia. Leukoplakia, the most frequent lesion, is defined as a white patch of oral mucosa that cannot be clinically or histologically classified as another disease. Erythroplakia is less common but carries a substantially higher risk of malignant transformation and is frequently associated with high-grade dysplasia or carcinoma in situ at diagnosis [3,4,5,6,7]. Other WHO-recognized OPMDs include oral lichen planus, oral submucous fibrosis, actinic cheilitis, and lupus erythematosus, all of which exhibit variable malignant potential [6,7,8].

The progression from premalignant lesions to OSCC is a multistep, prolonged process involving chronic inflammation, oxidative stress, and immune dysregulation. Interleukin-6 (IL-6), a pleiotropic pro-inflammatory cytokine, regulates cell proliferation, angiogenesis, apoptosis inhibition, and immune responses, and is implicated in the initiation and progression of various cancers, including OSCC [9,10,11,12]. Saliva, as a readily accessible and non-invasive biological fluid, is a promising medium for identifying biomarkers relevant to oral disease diagnosis and monitoring [13,14]. However, data on the relationship between salivary IL-6 levels and dysplasia grade or tumor stage remain inconsistent.

IL-6 is produced primarily by monocytes, macrophages, and locally activated immune cells and signals through membrane-bound and soluble receptors, enabling classical and trans-signaling pathways. Although levels are typically low in healthy individuals, IL-6 increases with aging, systemic inflammation, metabolic disorders, and chronic diseases [9,10]. In the oral environment, IL-6 is closely linked to periodontal inflammation, correlating with clinical attachment loss and disease severity and decreasing after periodontal therapy. Periodontal pathogens such as Porphyromonas gingivalis may further stimulate IL-6 production through STAT3-mediated immune activation, contributing to tissue destruction and bone resorption. These findings highlight the importance of controlling periodontal and systemic inflammatory conditions when evaluating IL-6 as a salivary biomarker [9,15,16].

In oral carcinogenesis, IL-6 links chronic inflammation to malignant transformation. Persistent exposure to inflammatory stimuli, including tobacco, alcohol, and periodontal pathogens, promotes sustained IL-6 production and a pro-tumorigenic microenvironment. Through activation of JAK/STAT3, MAPK, and PI3K/Akt pathways, IL-6 enhances tumor cell proliferation, survival, angiogenesis, and immune evasion [10,17,18]. Elevated IL-6 levels in saliva, serum, and tumor tissues are associated with tumor stage, histological grade, and poor prognosis, supporting its investigation as a non-invasive biomarker for early detection and risk stratification [11,12,18].

OSCC has a multifactorial etiology involving behavioral, environmental, infectious, and host-related factors. Tobacco use and alcohol consumption remain the principal risk factors in Europe, acting synergistically and accounting for up to 70–80% of head and neck squamous cell carcinomas [6,7,18]. Their carcinogenicity is attributed to polycyclic aromatic hydrocarbons and tobacco-specific nitrosamines in tobacco and to acetaldehyde generated during ethanol metabolism. Additional risk factors include betel quid chewing, high-risk human papillomavirus infection, nutritional deficiencies, immune suppression, occupational exposure to dust and heavy metals, and hereditary disorders such as Fanconi anemia and dyskeratosis congenita. Chronic exposure to these stimuli promotes genetic and epigenetic alterations, dysregulation of oncogenic pathways, and remodeling of the tumor microenvironment, facilitating progression from OPMDs to invasive carcinoma [6,18].

Tobacco smoke contains numerous carcinogens, including 4-(methylnitrosamino)-1-(3-pyridyl)-1-butanone (NNK) and N’-nitrosonornicotine (NNN), which can induce DNA damage and mutations in tumor suppressor genes, particularly tumor protein 53 (TP53). These mutations disrupt cell cycle regulation and apoptosis, facilitating malignant transformation of oral epithelial cells. Nicotine, the principal psychoactive component of tobacco, exerts its effects by binding to nicotinic acetylcholine receptors (nAChRs), activating signaling pathways involved in cell proliferation and survival. After absorption, mainly through the lungs, oral mucosa, and skin, nicotine is metabolized primarily in the liver to cotinine, which accounts for approximately 70–80% of total nicotine metabolism [19,20,21]. Measurement of salivary cotinine allows for objective assessment of individual exposure to tobacco smoke, which is closely linked to the accumulation of genetic alterations, including TP53 mutations, that drive the initiation and progression of OSCC [21]. Cotinine also has a longer half-life than nicotine, reaching up to 20 h, and is widely used as a biomarker of tobacco exposure in clinical and epidemiological studies [20].

Cotinine can influence biological processes related to cancer progression. Studies indicate that cotinine may modulate endothelial cell function, affect nitric oxide production, and enhance vascular endothelial growth factor (VEGF) expression, suggesting potential roles in angiogenesis and tumor progression [21]. Assessing cotinine alongside IL-6 may clarify interactions between tobacco exposure, inflammation, and malignant transformation.

Importantly, a substantial subset of OSCC arises in individuals without traditional behavioral risk factors. Non-smoking, non-drinking (NSND) patients may represent approximately 30–35% of OSCC cases and appear to constitute a distinct clinical subgroup, often characterized by older age, female predominance, and tumors located on the oral tongue [22]. The occurrence of OSCC in NSND individuals highlights alternative mechanisms, including genetic susceptibility, immune dysregulation, microbiome alterations, and chronic mucosal inflammation independent of tobacco and alcohol exposure. Recognition of this subgroup underscores the importance of detailed characterization of smoking and alcohol exposure—including smoking status, cumulative exposure (pack-years), and duration of abstinence—when interpreting biomarkers such as salivary IL-6 and cotinine, as inadequate stratification may obscure biologically distinct pathways of oral carcinogenesis [22,23].

Considering the multistage progression of premalignant lesions to OSCC, which may span several years, direct evaluation of lesion transformation in longitudinal studies is challenging. Therefore, the aim of this study was to evaluate salivary concentrations of IL-6 and cotinine in patients with premalignant lesions and OSCC, and to analyze correlations with dysplasia grade, tumor differentiation, disease stage, and exposure to risk factors such as tobacco. This combined assessment of inflammatory and tobacco exposure markers may provide a more comprehensive understanding of the mechanisms driving oral carcinogenesis and support the identification of high-risk patients. This approach may contribute to a better understanding of the diagnostic and prognostic value of these biomarkers in oral carcinogenesis.

## 2. Materials and Methods

The study was conducted at the Clinic of Maxillofacial Surgery at the Medical University of Silesia (SUM) in Katowice between 2014 and 2017. Among 267 patients diagnosed based on histopathological examination with OPMDs (study group I) or OSCC (study group II), 47 patients fulfilling the inclusion criteria were selected for the study. The sample size was determined by the availability of eligible participants during the study period.

Eligible participants were adults (≥18 years) with histopathologically confirmed OPMDs or OSCC and without clinical evidence of odontogenic or non-odontogenic oral infections. To minimize the influence of inflammatory confounders on salivary biomarker levels, individuals with periodontal disease or other active inflammatory lesions of the oral cavity were not eligible. Periodontal status was assessed using the Community Periodontal Index of Treatment Needs (CPITN), and only patients with a CPITN score of 0 were included.

Participants were excluded if they had systemic conditions known to affect inflammatory status or salivary biomarkers, including autoimmune or rheumatic diseases (e.g., rheumatoid arthritis), diabetes mellitus, cardiovascular diseases (including atrial fibrillation), chronic respiratory diseases, or obesity. Individuals receiving medications known to alter salivary flow or composition were also excluded, including antihistamines, antihypertensives, antidepressants, psychotropic agents, and bronchodilators. These medications may induce xerostomia, modify salivary protein composition, and influence cytokine expression, thereby potentially confounding the measurement of salivary IL-6 and cotinine levels. Additional exclusion criteria comprised leukocytosis and elevated inflammatory markers, defined as C-reactive protein (CRP) > 5 mg/L or procalcitonin > 0.05 ng/mL, as determined by laboratory testing and complete blood counts.

Participants were allocated into two study groups and one control group. Group I included 18 patients with OPMDs, group II included 29 patients with OSCC, and the control group (group III) comprised 40 individuals without OPMDs or OSCC who met the general study criteria. 

The control group consisted of individuals without pathological changes in the oral cavity or periodontal tissues (CPITN = 0) and who reported no history of smoking or alcohol consumption.

Unstimulated whole saliva was collected from each participant prior to incisional biopsy for histopathological verification, between 8:00 and 11:00 a.m., to reduce circadian variability. Patients were instructed to avoid eating, drinking, tooth brushing, smoking, and chewing gum prior to saliva collection.

Saliva was collected using single-use Salivette devices (Sarstedt, Nümbrecht, Germany). Patients chewed a sterile cotton swab for 30–45 s, after which the soaked swab was placed in a perforated insert and enclosed in a centrifuge tube for transport to the laboratory. Samples were centrifuged at 3000× *g* for 15 min to obtain a clear saliva supernatant (approximately 0.7 mL). The supernatant was aliquoted and stored at −80 °C in a DAIHAN Fre25-86 UniFreez™ ULT personal freezer prior (DAIHAN Scientific Co., Ltd., Wonju, Republic of Korea) to transport to the Department of Medical and Molecular Biology at the Medical University of Silesia for further analysis. To preserve biomarker stability, samples were thawed only once and were not subjected to repeated freeze–thaw cycles before analysis.

Salivary interleukin-6 (IL-6) concentrations were measured using commercial enzyme-linked immunosorbent assay (ELISA) kits (Bender MedSystems GmbH, Vienna, Austria) in accordance with the manufacturer’s instructions. Salivary cotinine levels were determined using the Salivary Cotinine Quantitative Enzyme Immunoassay Kit (Salimetrics LLC, State College, PA, USA). Absorbance was measured using a µQuant microplate reader (BioTek, Winooski, VT, USA), and data were processed with KCJunior 3.0 software (BioTek, USA). The assay sensitivity was 0.2 pg/mL for IL-6 and 0.15 ng/mL for cotinine, with an imprecision of 5.2% for both assays. Internal assay controls and calibration standards were included in each analytical run to ensure measurement accuracy and reproducibility. IL-6 and cotinine levels were analyzed according to the degree of dysplasia in the OPMDs group (grades I–III), histopathological differentiation of OSCC (G1–G4), tumor stage (T1–T4, TNM classification), smoking (<5 pack-years vs. >5 pack-years), and alcohol consumption (<10 years vs. >10 years).

Statistical analysis was performed using the Shapiro–Wilk test to assess normality of distribution. Intergroup comparisons were conducted using the Mann–Whitney U test or Kruskal–Wallis test, as appropriate, and correlations were evaluated using Spearman’s rank correlation coefficient (rho). Statistical significance was set at *p* < 0.05, *p* < 0.01, and *p* < 0.001. The study was approved by the Bioethics Committee of SUM (approval number KNW/0022/KB1/40/14, dated 1 July 2014), and all participants provided written informed consent prior to enrollment.

## 3. Results

Group I (OPMDs) included 18 patients (10 females, 8 males; aged 33–68 years, mean 55.5), and Group II (OSCC) included 29 patients (11 females, 18 males; aged 47–83 years, mean 66.8). Group III (controls) comprised 40 individuals without OPMDs or OSCC. Detailed descriptive statistics are presented in Table 1.

Grade I epithelial dysplasia was the most frequent histopathological finding in Group I, while moderately differentiated (G2) tumors predominated in Group II. The control group included individuals without pathological changes in the oral mucosa or periodontal tissues. Active cigarette smoking at the time of examination was reported by 16.7% of patients in Group I and 55.2% of patients in Group II, demonstrating significant intergroup differences (*p* < 0.05). In contrast, none of the control participants reported active smoking. Patients with OPMDs more frequently reported lower smoking intensity and a smoking duration of <5 years, whereas patients with OSCC more commonly reported ≥5 pack-years and a smoking history exceeding 5 years.

Similarly, long-term alcohol consumption (>10 years) was significantly more frequent in the OSCC group compared with both the OPMDs and control groups (*p* < 0.05). No alcohol use was reported in the control group.

Detailed smoking and alcohol histories were obtained, including smoking status, pack-years, and duration of alcohol use, to support interpretation of salivary cotinine and IL-6 levels and to account for non-smoking, non-drinking OSCC cases.

Mean salivary IL-6 and cotinine concentrations are summarized in Table 2. The highest mean levels of IL-6 (110.4 ± 20.4 pg/mL) and cotinine (66.3 ± 52.5 ng/mL) were observed in OSCC patients, while the lowest values were recorded in controls (12.7 ± 3.4 pg/mL and 2.6 ± 0.88 ng/mL, respectively). Intermediate levels were observed in OPMDs patients.

Statistical comparisons of IL-6 and cotinine between groups are presented in Table 3 and Table 4, showing significant differences for both biomarkers across all pairwise group comparisons (*p* < 0.05 to *p* < 0.0001). Furthermore, Cohen’s d was calculated to report effect sizes, revealing large to very large magnitudes for all observed differences, which confirms the practical significance of these findings.

**Figure 1:**
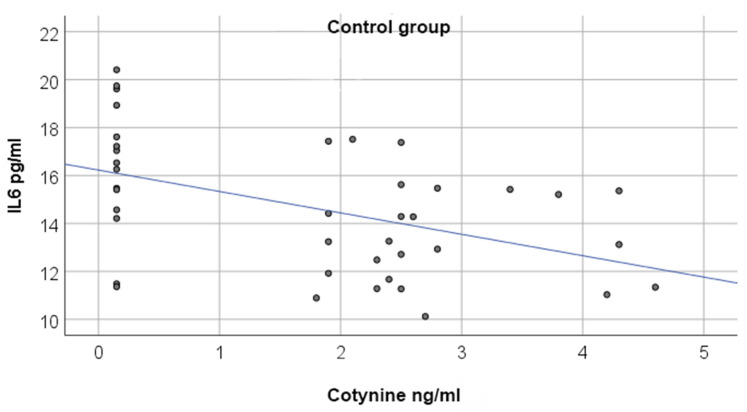
Illustration of the relationship between cotinine and IL-6 in the control group.

Spearman correlation analysis showed a moderate, negative correlation between cotinine and IL-6 in the control group (rho = −0.433, *p* = 0.005), whereas no significant correlation was found in either study group (Table 5, Figure 1).

Among OPMDs patients, Mann–Whitney U tests indicated significantly higher cotinine levels in active smokers compared to non-smokers (*p* = 0.008), while IL-6 concentrations were not significantly affected by smoking status (Table 6, Table 7 and Table 8). Similar patterns were observed in OSCC patients, with smokers exhibiting significantly higher cotinine levels (*p* < 0.001), but no significant differences in IL-6 (Table 9, Table 10 and Table 11).

**Figure 2:**
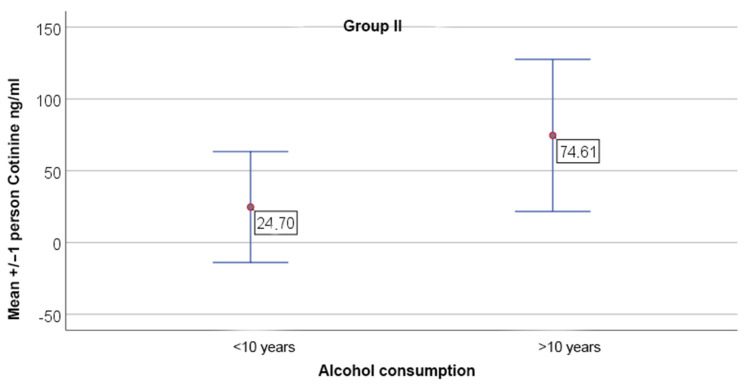
Comparison of cotinine levels between patients consuming high-proof alcohol for more than 10 years and those consuming for less than 10 years in study group II (OSCC). Data are presented as mean +/− SD. Significant differences were observed between the subgroups, *p* = 0.039.

**Figure 3:**
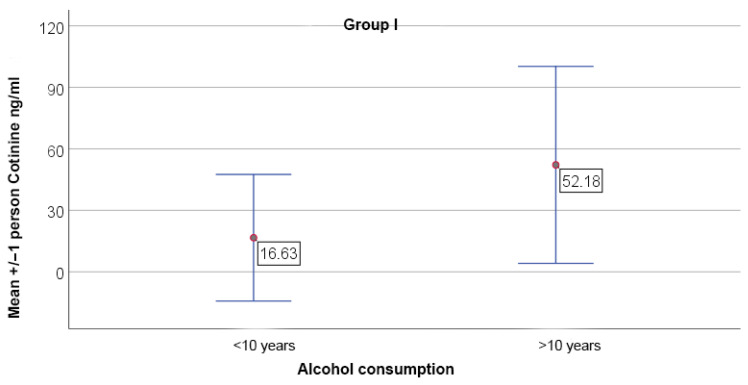
Comparison of cotinine levels between patients consuming high-proof alcohol for more than 5 years and those consuming for less than 10 years in study group I (OPMDs). Data are presented as mean +/− SD. Significant differences were observed between the subgroups, *p* = 0.385.

Long-term alcohol consumption (>10 years) was associated with elevated cotinine levels in both OPMDs and OSCC groups, while IL-6 levels remained unaffected (Figure 2 and Figure 3).

Other variables, including histopathological grade and TNM stage, did not show consistent or significant associations with IL-6 or cotinine.

## 4. Discussion

OSCC remains a major global health concern, ranking as the sixth most common cancer worldwide. The highest incidence is observed in South Asia, particularly in India, which accounts for approximately one-third of all global cases [24,25]. Globally, malignancies of the lip and oral cavity constitute roughly 2% of all cancers and occur nearly twice as frequently in males as in females, reflecting higher exposure to established risk factors such as tobacco use and alcohol consumption [25,26]. While HPV-positive oropharyngeal cancers continue to rise in certain populations, a gradual decline in oral cavity cancer incidence has been reported in some regions, suggesting that improvements in public health measures and early detection may be beginning to influence disease patterns [25,26]. Nevertheless, in low- and middle-income countries, limitations in screening and rapid diagnostic tools remain a significant barrier, often delaying intervention [27].

Several biomarkers from serum, oral tissues, and saliva have been proposed for early diagnosis and prognosis of OSCC; however, their utility is often constrained by low sensitivity, high false-positive rates, and a lack of large-scale validation [28,29]. Among these, IL-6 has emerged as a key biomarker, particularly in saliva. Early international work by Selvam et al. demonstrated significantly elevated salivary IL-6 in patients with oral leukoplakia and OSCC compared with healthy controls [29]. Subsequent studies have confirmed elevated IL-6 levels in OSCC relative to OPMDs, with additional associations observed with IL-6 gene polymorphisms and correlations with tumor stage, histological grade, and disease aggressiveness [29,30,31,32,33].

In our cohort, we observed statistically significant elevations of IL-6 in OSCC patients compared to both OPMDs and controls (*p* < 0.05), consistent with prior reports. Interestingly, while IL-6 levels in OPMDs were intermediate, differences between mild and moderate dysplasia were not statistically significant, highlighting potential limitations of IL-6 in distinguishing early dysplastic stages. These findings align with Sharma et al., who reported higher IL-6 in severe dysplasia compared with milder forms, while also suggesting that larger sample sizes are required to robustly capture these differences [34].

Mechanistically, IL-6 contributes to OSCC pathogenesis through paracrine and autocrine signaling, modulation of immune responses, and promotion of tumor growth and invasiveness [33,35,36,37,38]. Elevated IL-6 secretion by tumor cells and monocytes may induce local immunotolerance, facilitating malignant progression. Evidence from other solid tumors and hematologic malignancies further supports the diagnostic and prognostic relevance of IL-6 [39,40]. In our study, IL-6 levels were largely independent of active smoking or alcohol consumption, reinforcing the concept that IL-6 elevations primarily reflect malignant transformation rather than acute lifestyle exposures.

Recent evidence further indicates that metabolic reprogramming within the tumor microenvironment plays a critical role in cancer progression. Lactate, traditionally regarded as a glycolytic byproduct, is now recognized as a regulator of tumor metabolism, immune suppression, and gene expression through protein lactylation. This metabolic–inflammatory crosstalk promotes tumor proliferation, immune evasion, and microenvironment remodeling, processes that may synergize with IL-6-mediated signaling to sustain a pro-tumorigenic milieu in OSCC [41].

In the oral cavity, microbiome dysbiosis may further contribute to lactate accumulation through bacterial fermentation, thereby reinforcing local acidification, metabolic reprogramming, and inflammatory signaling pathways that support tumor initiation and progression.

Together, these findings support an integrated model in which inflammatory cytokines such as IL-6, metabolic alterations including lactate accumulation, and oral microbiome dysbiosis interact to shape a tumor-promoting microenvironment and drive oral carcinogenesis.

Cotinine, a complementary biomarker, objectively reflects tobacco exposure, encompassing both active and passive smoking, which remain major modifiable risk factors for OSCC [42,43,44]. In line with expectations, cotinine levels were significantly higher among active smokers in both OPMDs and OSCC groups (*p* < 0.01), demonstrating its utility for monitoring patient adherence to behavioral interventions. The combination of IL-6 and cotinine may thus provide a multidimensional perspective: IL-6 as a marker of cellular transformation and disease severity, and cotinine as a real-time indicator of exposure to carcinogens. This approach aligns with the concept of field cancerization, where persistent exposure increases the likelihood of synchronous and metachronous lesions [45,46,47].

The risk of second primary tumors remains a significant clinical concern in OSCC. Population-based studies report cumulative 5-year incidences ranging from 13% to 30%, most frequently affecting the head and neck region or lungs [45,46,47]. Our findings reinforce these observations: patients with higher tobacco exposure, as reflected by salivary cotinine, may be at elevated risk, emphasizing the clinical relevance of integrating biomarker monitoring with lifestyle counseling and routine surveillance.

These observations reinforce the potential of salivary IL-6 and cotinine as complementary, non-invasive biomarkers for evaluating oral epithelial transformation and tobacco exposure. The differences observed between groups, in line with prior studies, suggest that these markers may aid in risk stratification and early detection, particularly in high-risk populations. However, the variability in IL-6 across dysplasia grades and its independence from acute lifestyle exposures indicate that further longitudinal studies are needed to clarify its temporal dynamics relative to malignant progression. Multi-center investigations could help define standardized thresholds and assess the performance of combined biomarker panels, ultimately refining diagnostic and prognostic precision in OSCC management.

### Limitations

This study has several limitations that should be considered when interpreting the findings. First, the sample size was relatively small and determined by the availability of eligible participants during the study period rather than by formal sample size calculation. Although strict inclusion criteria improved internal validity, they limited recruitment and reduced statistical power, particularly in subgroup analyses. For example, the proportion of active smokers in the OPMDs group was low (16.7%, n = 3), which substantially limits the reliability of comparisons between smokers and non-smokers. Consequently, subgroup analyses should be considered exploratory, and the absence of statistically significant differences in IL-6 levels across exposure categories may reflect insufficient power rather than true biological independence.

Second, the cross-sectional design precludes causal inference and limits the ability to assess temporal relationships between IL-6 levels, tobacco exposure, and malignant transformation. Longitudinal studies are required to determine whether elevated salivary IL-6 precedes disease progression or reflects established pathological changes.

Third, the study was conducted at a single tertiary care center, which may restrict the generalizability of the findings to broader populations with different demographic, environmental, or behavioral risk profiles. Multicenter studies are needed to validate these results and establish clinically relevant biomarker thresholds.

Fourth, although extensive measures were implemented to minimize inflammatory confounding—including exclusion of individuals with systemic diseases, leukocytosis, elevated CRP or procalcitonin, and periodontal disease (CPITN = 0)—salivary IL-6 remains a marker of the general inflammatory state and may be influenced by factors not fully controlled in this study. Subclinical inflammation, undetected periodontal microinflammation, obesity-related low-grade inflammation, psychological stress, circadian rhythm variability, and age-related immune changes may all affect IL-6 concentrations. These influences should be considered when interpreting salivary IL-6 as a biomarker of oral carcinogenesis.

Fifth, multivariate analyses were not performed due to the limited sample size. As a result, potential interactions between risk factors—such as tobacco exposure, alcohol use, age, and inflammatory status—could not be fully evaluated. Future studies with larger cohorts should employ multivariable models to clarify independent predictors of IL-6 elevation and OSCC risk.

Finally, although smoking exposure was characterized using self-reported history and salivary cotinine, residual misclassification remains possible, particularly regarding passive smoke exposure and long-term cessation effects. More detailed exposure assessment and repeated biomarker measurements could improve exposure classification and strengthen causal interpretation.

## 5. Conclusions

Salivary cotinine reliably reflected tobacco exposure, with higher levels observed in OSCC patients, particularly in advanced TNM stages, and in both pre-malignant and malignant lesions among smokers. Salivary IL-6 was elevated in OSCC compared to OPMDs and controls and was significantly higher in patients with long-term alcohol consumption. IL-6 levels did not correlate with dysplasia grade or tumor differentiation. These findings support the potential utility of salivary IL-6 and cotinine as non-invasive biomarkers for assessing oral epithelial transformation and exposure to key risk factors.

## Figures and Tables

**Table 1:** Clinical and behavioral characteristics of the study groups.

Variable	Group I: OPMDs (n = 18)	Group II: OSCC (n = 29)	Group III: Controls (n = 40)	*p*-Value
Age, years	33–68 (mean 55.5)	47–83 (mean 66.8)	18–37 (mean 26.4)	—
Sex, n (%)	Female: 9 (50.0%) Male: 9 (50.0%)	Female: 11 (37.9%) Male: 18 (62.1%)	Female: 25 (62.5%) Male: 15 (37.5%)	—
Active smoking, n (%)	3 (16.7%)	16 (55.2%)	0 (0%)	<0.05
High-proof alcohol consumption, n (%)	6 (33.3%)	15 (51.7%)	0 (0%)	<0.05
Smoking exposure (pack-years), n (%)	<5: 15 (83.3%) ≥5: 3 (16.7%)	<5: 13 (44.8%) ≥5: 16 (55.2%)	0	<0.05
Alcohol consumption duration, n (%)	<10 years: 12 (66.7%) ≥10 years: 6 (33.3%)	<10 years: 14 (48.3%) ≥10 years: 15 (51.7%)	0	<0.05
Histopathological diagnosis	Grade I: 13 (72.2%) Grade II: 4 (22.2%) Grade III: 1 (5.6%)	G1: 6 (20.7%) G2: 19 (65.5%) G2 + metastasis: 2 (6.9%) G3: 2 (6.9%)	Not applicable	—

**Table 2:** Mean ± SD of IL-6 and cotinine concentrations in the control group, study group I (OPMDs), and study group II (OSCC).

	Cotinine [ng/mL]	IL6 [pg/mL]
Control group (mean ± SD)	2.6 ± 0.88	12.7 ± 3.4
Group I (mean ± SD	24.2 ± 31.3	22.8 ± 4.5
Group II (mean ± SD)	66.3 ± 52.5	110.4 ± 20.4

**Table 3:** Comparison of mean IL-6 values among the control group, study group I (OPMDs), and study group II (OSCC). Data in cells represent *p*-values and Cohen’s d effect sizes (in parentheses).

IL6 [pg/mL]	Control Group	Group I	Group II
Control group	x	*p* < 0.0001 (d = 2.53)	*p* < 0.0001 (d = 6.69)
Group I	*p* < 0.0001 (d = 2.53)	x	*p* < 0.0001 (d = 5.98)
Group II	*p* < 0.0001 (d = 6.69)	*p* < 0.0001 (d = 5.98)	x

**Table 4:** Comparison of mean cotinine values among the control group, study group I (OPMDs), and study group II (OSCC). Data in cells represent *p*-values and Cohen’s d effect sizes (in parentheses).

Cotinine [ng/mL]	Control Group	Group I	Group II
Control group	x	*p* < 0.05 (d = 0.97)	*p* < 0.0001 (d = 1.71)
Group I	*p* < 0.05 (d = 0.97)	x	*p* < 0.01 (d = 0.95)
Group II	*p* < 0.0001 (d = 1.71)	*p* < 0.01 (d = 0.95)	x

**Table 5:** Correlations between cotinine and IL-6 in the individual study groups.

Statistical Factor	Control Group	Group I	Group II
rho	−0.433	0.181	0.280
*p*	0.005	0.473	0.141

**Table 6:** Comparison of smokers and non-smokers in study group I (OPMDs) regarding IL-6 levels.

	Non-Smokers IL-6 pg/mL	Smokers IL-6 pg/mL
M ^1^	26.98	27.86
Me ^2^	26.75	30.19
SD ^3^	3.80	7.11

^1^ M—mean; ^2^ Me—median; ^3^ SD—standard deviation.

**Table 7:** Comparison of smokers and non-smokers in study group I (OPMDs) regarding cotinine levels.

	Non-Smokers Cotinine ng/mL	Smokers Cotinine ng/mL
M	14.91	106.66
Me	3.50	102.34
SD	16.71	10.78

**Table 8:** Comparison of U Mann–Whitney test in study group I regarding IL-6 and cotinine levels.

Cotinine ng/mL	Z	*p*
IL-6 pg/mL	−0.652	0.514
Cotinine ng/mL	−2.667	0.008

**Table 9:** Comparison of smokers and non-smokers in study group II (OSCC) regarding IL-6 levels.

	Non-Smokers IL-6 pg/mL	Smokers IL-6 pg/mL
M	97.0	100.14
Me	99.39	98.76
SD	26.14	23.39

**Table 10:** Comparison of smokers and non-smokers in study group II (OSCC) regarding cotinine levels.

	Non-Smokers Cotinine ng/mL	Smokers Cotinine ng/mL
M	22.28	97.73
Me	2.85	98.76
SD	39.66	34.04

**Table 11:** Comparison of U Mann–Whitney test in study group II regarding IL-6 and cotinine levels.

Cotinine ng/mL	Z	*p*
IL-6 pg/mL	−0.257	0.797
Cotinine ng/mL	−3.334	0.001

## Data Availability

The original contributions presented in this study are included in the article. Further inquiries can be directed to the corresponding author.

## Abbreviations

The following abbreviations are used in this manuscript:

CPITN	Community Periodontal Index of Treatment Needs
CRP	C-reactive protein
IL-6	Interleukin-6
M	mean
Me	median
NNK	4-(Methylnitrosamino)-1-(3-pyridyl)-1-butanone
NNN	N’-Nitrosonornicotine
nAChRs	Nicotinic acetylcholine receptors
OPMDs	Oral potentially malignant disorders
OSCC	Oral squamous cell carcinoma
SD	standard deviation
SUM	Medical University of Silesia
TP53	Tumor protein 53
VEGF	Vascular endothelial growth factor
